# PCSK9 Contributes to the Cholesterol, Glucose, and Insulin2 Homeostasis in Seminiferous Tubules and Maintenance of Immunotolerance in Testis

**DOI:** 10.3389/fcell.2022.889972

**Published:** 2022-05-02

**Authors:** R.-Marc Pelletier, Hamed Layeghkhavidaki, Nabil G. Seidah, Annik Prat, María L. Vitale

**Affiliations:** ^1^ Department of Pathology and Cell Biology, Université de Montréal, Montreal, QC, Canada; ^2^ Biochemical Neuroendocrinology Laboratory, Montreal Clinical Research Institute (IRCM), Montreal, QC, Canada

**Keywords:** testis, Sertoli cell, germ cell, cholesterol, insulin, glucose, type 2 diabetes, cytokines

## Abstract

The PCSK9 contribution to cholesterol and immunotolerance homeostasis and response to glucose, and insulin in testis and hypophysis were studied using *Pcsk9*-deficient (^−/−^) and transgenic [Tg (PCSK9)] mice, and diabetic, obese *ob/ob* and *db/db* mice. The spermatids/spermatozoa acrosome, peritubular vessels, and epididymal adipocytes were PCSK9- and LDL-R-positive. The pro-PCSK9/PCSK9 ratio was high in interstitial tissue-fractions (ITf) and spermatozoa and low in seminiferous tubule-fractions (STf) in normal adult mice. This ratio decreased in ITf in *ob/ob* and *db/db* mice but increased in tubules in *ob/ob* mice. Deleting *pcsk9* lowered cholesterol in serum but increased testicular cholesterol. Furthermore, HMGCoA-red, ACAT-2 and LDL-R turnover increased whereas SR-BI decreased in ITf; in tubules, ABCA1 decreased and 160 kDa LDL-R increased in *Pcsk9* ^−/−^ mice. Excess testicular cholesterol could result from increased cholesterol synthesis and uptake with reduction in SR-BI-mediated efflux in ITf and from the overload of apoptotic cells, lowered ABCA1-mediated efflux and stimulated LDL-R protein synthesis in tubules in *Pcsk9* ^−/−^ mice. Concomitantly with the cholesterol accumulation, tubules showed infiltrates of immune cells, elevated IL-17A and IL-17RA, and changes in the immunotolerance homeostasis. PCSK9 deficiency decreased glucose in tubules and spermatozoa while increasing insulin2 in ITf and tubules not serum. Moreover, IR-α, and IR-β augmented in tubules but decreased in the anterior pituitary; IR-α increased whereas IR-β decreased in ITf. The histology and cholesterol levels were normal in Tg (PCSK9) mouse testis. The excess cholesterol creates a milieu favorable to the action of high IL-17A and IL-17RA, the development of inflammatory conditions and self-tolerance breakdown in testis.

## Introduction

Alterations in cholesterol and lipid metabolism (Pasquali et al., 2007) combined with obesity have been associated with infertility in men (Campbell et al., 2015). The incidence of dyslipidemia is elevated in infertile men (Ramírez-Torres et al., 2000). Spermatozoa from subjects with idiopathic infertility exhibit high cholesterol to phospholipid ratios (Sugkraroek et al., 1991). Yet, no relation was found between the cholesterol in the circulation and the cholesterol in spermatozoa, semen or seminal plasma (Travis and Kopf, 2002) though cholesterol levels and fertility are negatively correlated in human spermatozoa (Keber et al., 2013). Here, the present study advances the concept that cholesterol homeostasis is regulated by local factors in the male gonad. The contribution of the proprotein convertase subtilisin/kexin type 9 (PCSK9) in the synthesis, uptake, and efflux of cholesterol and in the immunotolerance in the mouse testis is assessed.

PCSK9 is an enzyme of the proteinase K subfamily of subtilisin-related serine endopeptidases (Abifadel et al., 2003; Maxwell and Breslow, 2004) that contributes to the cholesterol homeostasis by reducing the number of low density lipoprotein (LDL) receptors (LDL-R) on the cellular membrane and the cell’s ability to take up LDL (Seidah et al., 2003). PCSK9 acts as a natural inhibitor of the LDL-R pathway by targeting the receptor to the lysosomal pathway for degradation (Maxwell and Breslow, 2004). Deleting *pcsk9* increases the amount of LDL-R protein in the mouse liver and results in decreased plasma cholesterol levels (Rashid et al., 2005; Zaid et al., 2008). This study assesses the LDL-R response in the testis to the deletion and overexpression of *pcsk9*.

In recent years, the incidence of metabolic disorders and insulin-dependent type2 diabetes reached epidemic proportions in adolescents and young subjects of reproductive age (Scully, 2012). The present study evaluates the consequences of deleting and over expressing the *pcsk9* gene on elements of the glucose metabolism that influence the signaling pathways activated by insulin receptors in the testis and hypophysis.

PCSK9 is expressed principally in liver, ileum, jejunum and, in lesser amounts, in kidney, cerebellum, thymus, testis, tail of epididymis (Seidah et al., 2003; Zaid et al., 2008) and pancreas (Da Dalt et al., 2018; Peyot et al., 2021). After the removal of the signal peptide, the resulting 75-kDa pro-protein or proPCSK9 is cleaved at the N-terminal releasing a 13 kDa pro-domain peptide that non-covalently binds to 62 kDa PCSK9, the mature form of the convertase (Seidah et al., 2003). The autocatalytic cleavage removes the inhibitory prodomain which allows PCSK9 to breakout from the endoplasmic reticulum (Benjannet et al., 2004). The released 13 kDa + 62 kDa heterodimer can be secreted in the blood circulation (Benjannet et al., 2004; McNutt et al., 2007). Another extracellular form of PCSK9 in addition to the heterodimer is the 62 kDa-PCSK9 and a 55 kDa-PCSK9 which results from a second proteolytic cleavage by the protease furin (Benjannet et al., 2006). Two forms are intracellular: 75 kDa-pro PCSK9 and 62 kDa-PCSK9. In tissues, PCSK9 was thought to be expressed by cells “that have the capacity to proliferate and differentiate” (Seidah et al., 2003). Cell proliferation and differentiation are prevailing in the testis. Northern blotting identified a smaller sized 2.2-kb PCSK9 mRNA in whole testis extracts compared to 2.8-kb in other tissues (Seidah et al., 2003) though the physiological significance of this reduced size is unknown. The nature/state of PCSK9 is different in the serum and tissues.

In testis, lipids and endogenous substrates supply two thirds of the energy produced aerobically (Free et al., 1969); the glucose provided by spermatocytes and spermatids contributes the remaining third (Tepperman, 1950) through the EMbden-Meyerh path of glycolysis, acetyl CoA formation, and the citric acid cycle (Hollinger and Davis, 1968). To identify the contribution of PCSK9 in the cholesterol and glucose-insulin metabolism in the testis, spermatozoa and anterior hypophysis, we took advantage of the *pcsk9*-deficient (^−/−^) mice and *pcsk9* transgenic [Tg (PCSK9)] mice over expressing Pcsk9 (Zaid et al., 2008). In addition, to evaluate the PCSK9 response to diabetes-induced high glucose/insulin in the testis, the two type 2 diabetes leptin-deficient (*ob/ob*) (Zhang et al., 1994) and leptin receptor-deficient (*db/db*) (Tartaglia et al., 1995) mouse models were used. The testis is made up of two anatomically distinct cellular compartments exerting different functions: the interstitium in which cholesterol serves principally in steroids production and the seminiferous tubules that host developing germ cells. For this reason, all measurements were carried separately in interstitial tissue- and tubule-enriched fractions rather than in whole testis extracts.

Cellular cholesterol is synthesized from acetyl-CoA and/or taken up from the ambient milieu or circulation. The free movement of LDL and high-density lipoproteins (HDL) in the blood through pores in capillaries enables the accumulation of cholesteryl esters by lipoproteins and steroid production in the interstitial tissue of the testis (Azhar and Menon, 1982; Fofana et al., 1996). In this location, cholesterol is synthesized *de novo* in Leydig cells (Payne and O'Shaughnessy, 1996) and up taken from the blood through HDL (Fofana et al., 1996) and LDL. The two mechanisms are coordinated; they are regulated by the amount of cholesterol present in the endoplasmic reticulum in the cell. Thus, blocking the synthesis of cholesterol augments its uptake *via* HDL/SR-BI and LDL/LDL-R. HDL transports cholesterol and phospholipids from peripheral tissues (including testis) back to the liver through the reverse cholesterol transport (Liscum and Munn, 1999). In seminiferous tubules, Sertoli cells have the capacity to synthesize cholesterol from acetate *in vitro* (Wiebe and Tilbe, 1979) though *in vivo*, the amount synthetised is small. The cholesterol substrate requirements exceed the Sertoli cell synthesis capacities. HDLs reduce the speed of cholesterol synthesis in Sertoli cells (Maboundou et al., 1995). In prepubertal cultured Sertoli cells (Fofana et al., 1996) and pubertal rat tubules, the basement membrane was said to allow the selective transfer of cholesterol from HDL and block entry of LDL (Fofana et al., 2000). It is worth noting that the development of the basement membrane in seminiferous tubules and the maturation of Sertoli cells are not completed during the neonatal period and puberty. The commitment of Sertoli cells occurs in adulthood. In contrast to the neonatal and pubertal testis, in the adult rodent testis, capillaries are made up of an uninterrupted layer of flat endothelial cells joined together by tight junctions and surrounded by a continuing basal lamina (Mayerhofer et al., 1989). The cholesterol taken up by LDL-R has access to Sertoli cells.

The present study reveals strong differences in the state of PCSK9 in the interstitium and seminiferous tubules. The pro-PCSK9/PCSK9 ratio was high in interstitial tissue and spermatozoa and low in tubules in normal adult mice suggesting enhanced autocatalytic cleavage of 75 kDa pro-PCSK9 in tubules. This ratio decreased in the interstitium in *ob/ob* and *db/db* mice but increased in tubules in *ob/ob* mice suggesting the stimulation of the 75 kDa pro-PCSK9 cleavage in the interstitial tissue and its hindrance in tubules. Deleting *pcsk9* lowered cholesterol in serum but increased testicular cholesterol. Concomitantly with the cholesterol excess, tubules showed an overload of apoptotic cells, infiltrates of immune cells, augmented IL-17A and IL-17RA, IR-α and IR-β subunits chains and changes in the immunotolerance homeostasis. Furthermore, the lack of PCSK9 decreased glucose concentrations in tubules and spermatozoa while increasing insulin2 levels in the interstitial tissue and tubules but not in plasma. In Tg (PCSK9) mice, the overexpression of the *pcsk9* gene restored spermatogenesis and normal histology of the testis and testicular cholesterol levels indicating that the tubule dysfunction was a consequence of the lack of PCSK9.

## Materials and Methods

### Chemicals

Diaminobenzidine tetrachloride from Sigma (St-Louis, MO, United States); phenylmethane-sulfonyl fluoride (PMSF), leupeptin, aprotinin, and Lumi-light^Plus^ chemiluminescence detection kit from Roche (Laval, QC, Canada); potassium bisperoxo (1,10-phenanthroline) oxovanadate (V) [bpV (phen)] from Calbiochem (San Diego, CA, United States) and horseradish peroxidase (HRP)-conjugated streptavidin from Molecular Probes (Eugene, OR, United States).

### Antibodies

Goat polyclonal anti-PCSK9 from R&D (Minneapolis, MN, United States), rabbit polyclonal anti-PCSK9 from Abclonal Science Inc. (Woburn, MA, United States), mouse monoclonal anti-IR-β subunit from Millipore (Etobicoke, ON, Canada), rabbit polyclonal anti-IR-α subunit from Biorbyt (Cambridge, United Kingdom); rabbit polyclonal anti LDL-R, rabbit polyclonal anti-ABCA1, purified rabbit polyclonal anti-SR-BI, rabbit polyclonal anti-SR-BII, rabbit polyclonal anti-IL-17 and rabbit polyclonal anti-LDL-R from Novus Biologicals (Littleton, CO, United States); rabbit polyclonal anti-ACAT-1 and anti-ACAT-2 from Cayman Chemical (Ann Arbor, MI); chicken polyclonal anti-hormone-sensitive lipase (HSL) from ProSci Inc. (Poway, CA, United States); and rabbit polyclonal anti-IL-17RA from Abcam (Cambridge, MA, United States); HRP-conjugated anti-mouse IgG, HRP-conjugated anti-rabbit IgG, HRP-conjugated anti-goat IgG, biotinylated anti-rabbit IgG from Jackson ImmunoResearch Laboratories Inc. (West Grove, PA, United States); HRP-conjugated anti-chicken IgG (H + L) made in goat from Vector Laboratories Inc. (Burlingame, CA, United States).

### Animals

#### Normal Mouse

Studies on postnatal development were carried out on the same 7- to >60-day-old male mice or >60 days adult of BALB/cJ background that we used and described earlier (Pelletier et al., 2015; Pelletier et al., 2018; Pelletier et al., 2020a; Pelletier et al., 2020b). Three animals were used per age group. Mice were housed at room temperature (RT) with food and water *ad libitum* and exposed to a 12 h: 12 h light dark cycle.

#### PCSK9 Knockout (^−/−^) and Transgene

[Tg (PCSK9)] mice Ten-weeks old *Pcsk9* ^−/−^ male mice that did not or did receive a transgene expressing PCSK9 and wild type (WT) of the same strain as control were used. All procedures were approved by the Clinical Research Institute of Montreal animal care committee. *Pcsk9* ^−/−^ mice lacking the *pcsk9* proximal promoter and exon 1 have been described as well as Tg (Pcsk9) mice over expressing Pcsk9 in hepatocytes under the ApoE promotor (Zaid et al., 2008).

#### Diabetic and Obese Mice

To identify the consequences of diabetes and obesity on PCSK9 protein expression we used the same spontaneously diabetic and obese *ob/ob* and *db/db* as the ones we used and described in our earlier studies (Pelletier et al., 2018; Pelletier et al., 2020a; Pelletier et al., 2020b). Ten male mice aged of 10 weeks with the leptin receptor (B6.BKS(D)-*Leprdb*/J homozygotes (*db*/*db*) Stock Number (00697) mutation, ten male mice aged of 10 weeks with the leptin (B6.Cg-*Lepob*/J homozygotes (*ob/ob*) Stock Number (00632) mutation both experimental group on the C57BL/6J genetic background and three WT mice were used. Mice were purchased from Jackson Lab (Bar Harbor, ME, United States). All procedures were approved by the Animal Care Committee of the Université de Montréal.

The total number of animals per experimental condition was five-to-nine. Western blots were run using three different animals per experimental condition. Different western blots of the same protein, but obtained with different animals, showed similar results. However due to the variability of the intensity of the immunoreactive bands on the films, the results from different blots were neither mixed nor normalized. Therefore, the results showed in the figures are the ones derived from a western blot run with samples from three animals per experimental condition even though the total number of animals per experimental condition is higher than three.

#### Isolation of Seminiferous Tubule-Enriched Fractions

The interstitium and the seminiferous tubules of the testis have different topography and functions. Therefore, all our assays were performed on interstitium- and seminiferous tubule-enriched fractions. In addition, to maximize detection of phosphorylated protein forms within the samples under study, tissue fraction were not pre-exposed to enzymes and processed as described earlier (Akpovi and Pelletier, 2009; Pelletier et al., 2015). Briefly, seminiferous tubules were mechanically teased apart from the interstitium from freshly decapsulated testes in cold phosphate buffered saline (PBS: 137 mM NaCl, 3 mM KCl, 8 mM Na_2_HPO_4_, 1.5 mM KH_2_PO_4_, pH 7.4) containing 2 mM PMSF, 1 mM EGTA, 2 μg/ml leupeptin, 2 μg/ml aprotinin, 4 mM Na_3_VO_4_, 80 mM NaF and 20 mM Na_4_P_2_O_7_ with 10 μM bpV (phen). The resulting seminiferous tubule-interstitium suspension was centrifuged 15 min at 400 rpm (GS-6R Beckman Centrifuge, JH-3.8 Rotor) at 4°C after having been allowed to decant. The interstitial tissue- (ITf) (supernatant) and seminiferous tubule-enriched (STf) (pellet) fractions were centrifuged 10 min at 1,000 rpm (GS-6R Beckman Centrifuge, JH-3.8 Rotor) at 4°C. Each fraction was characterized under the light microscope as described earlier (Pelletier et al., 2018; Akpovi and Pelletier, 2009).

#### Isolation of Epididymal Spermatozoa

The epididymal spermatozoa were obtained as described earlier (Akpovi et al., 2006). Briefly, epididymides were diced in cold PBS with proteases and phosphatase inhibitors, filtered through a 74 mm mesh, and centrifuged at 2,000 rpm for 15 min in a GS-6R Beckman centrifuge (JH-3.8 Rotor) at 4°C. Then, spermatozoa were recovered and resuspended 5 min in 10 mM Tris-HCl, pH 8, containing 1 mM EDTA to lyse epithelial and blood cells (Herrada and Wolgemuth, 1997), washed twice, and diluted 1:1 in cold PBS with proteases and phosphatase inhibitors. Cells were sonicated in a Fisher Sonic Dismembrator (model 300; Fisher, Farmington, NY) during three 30 s intervals.

#### Anterior Pituitary

After decapitation, the anterior lobe of the mouse pituitary gland was dissected free from the intermediate and posterior lobes. Anterior pituitaries were placed in PBS-protease and phosphatase inhibitors, sonicated and stored at −80°C until use as previously described (Vitale et al., 2017).

#### Protein Quantification

Proteins in samples were assayed using materials from BioRad (BioRad, Mississauga, ON, Canada).

#### Electrophoresis and Western Blot Analyses

Thirty µg total homogenate of sample were loaded on polyacrylamide gels, separated by 10% or 12% SDS-PAGE, transferred onto nitrocellulose membranes and subjected to western blotting as previously described (Vitale et al., 2009). In all western blot experiments, the membranes were first stained with Ponceau red to ensure equal loading. Briefly, membranes were blocked 1 h at 37°C with 5% skimmed milk in TRIS-buffered saline (TBS: 137 mM NaCl, 27 mM KCl, 25 mM Tris-HCl pH 7.4) then, incubated with the different antibodies. The antibody dilutions were prepared in 5% skimmed milk-TBS as follows: 1:500 for goat polyclonal anti-PCSK9 antibody, 1:500 for anti-ACAT-1, 1:250 for anti-ACAT-2, 1:500 for anti-HSL, 1:2,000 for anti-SR-BI, 1:1,000 for anti-SR-BII, 1:500 for anti-ABCA1, 1:250 for anti-IR-α, 1:800 for anti-IR-β, 1:2000 for IL-17, 1: 100 for anti-IL-17RA and 1:1500 for anti-LDL-R. Next, membranes were washed in TBS containing 0.05% Tween 20 and incubated 1 h with the corresponding secondary antibody conjugated to HRP at room temperature. The antigen-antibody complexes were detected by chemiluminescence. The intensity of the immunoreactive bands was quantified by laser scanning with the public Scion Image Software (Scioncorp, MD, United States).

#### Serum and Testicular Fraction Cholesterol Measurements

Free cholesterol (FC) and total cholesterol (TC) in interstitial tissue and seminiferous tubule-fractions (volume equivalent to 1 mg of total protein) and in serum (80–100 µl) were measured using the method we described in detail earlier (Akpovi et al., 2006). Briefly, tissue fraction homogenates and serum were extracted with hexane-isopropanol (3:2) and evaporated at 37°C with N-EVAP nitrogen evaporators (Organomation Associates, Inc. Berlin, MA, United States). An enzymatic kit (Wako Chemical USA, Richmond, VA, United States) was used to measure FC and TC. Esterified cholesterol (EC) was determined by subtracting FC from TC. FC and EC contents are expressed in mg of cholesterol/dl of serum or in mg of cholesterol/mg of total protein of enriched tissue fraction.

#### Serum Testosterone

Serum testosterone concentrations were measured by EIA with a Cayman Chemical (Cayman Chemical Company, Ann Arbor, MI, United States) enzyme immunoassay kit according to the manufacturer’s instructions. The method sensitivity was 6 pg/ml. All samples were assessed in duplicate in a single assay with coefficient of variation ranging from 0.85 to 7.27% between duplicates.

#### Serum and Tissue Glucose Measurements

Serum glucose concentrations were measured within 5 h following an overnight (18 h) fasting in mice fed a standard chow. Serum, ITf and STf glucose concentrations were measured with a colorimetric enzymatic (glucose-oxidize) assay (Mutarotase-GOD) (Autokit Glucose Wako, Wako, TX, United States) according to the manufacturer’s instructions. Tissue factions were prepared as described (Moraes et al., 2004) with modifications. Briefly, STf and ITf were sonicated in 6N perchloric acid while in an ice bath. The acid homogenates were centrifuged at 14,000 *g* and the supernatant used for glucose determination. 10 μl of serum or ITf or STf homogenates were mixed with 1.5 ml color reagent and incubated for 10 min. The absorbance of samples and standards was measured at 505 nm against the blank.

#### Serum and Tissue Insulin Measurements

Serum and tissue fraction insulin concentrations were measured with commercially available ELISAs as we described earlier (Pelletier et al., 2020a). Briefly, STf and ITf were homogenized with a tissue grinder in an acid ethanol solution (180 mM HCl in 70% ethanol; 0.01 ml/mg tissue) on ice (Vijay et al., 2008). Tissue lysates were sonicated (Fisher Sonic Dismembrator) 3 × 15 s and centrifuged 5 min at 10,000 *g* at 4°C. The supernatant was recovered for insulin determination. We measured total insulin (insulin T) which is insulin 1+ insulin2 levels using an ALPCO Diagnostics (Salem, NH, United States) ELISA kit. In addition, we used the Aviva Systems Biology Ins2 ELISA kit (San Diego, CA, United States) which measures only insulin2.

#### Morphological and Immunolabeling Studies

Testes were perfusion-fixed with Bouin’s fixative (Kabbaj et al., 2001; Kabbaj et al., 2003). For morphological studies tissue sections were stained with PAS. Immunolabeling Endogenous peroxidase activity was inhibited with 0.6% hydrogen peroxide (H_2_O_2_) in 70% ethanol (Pelletier et al., 1999). Unspecific binding was blocked with 0.5% skim milk in TBS containing 0.1% Tween-20 (TBST) for 1 h at 37°C. Next, tissue sections were incubated overnight at room temperature with either (1:50) rabbit polyclonal anti-PCSK9 or (1:1,000) the rabbit polyclonal anti LDL-R, then, 1 h with biotinylated anti-rabbit IgG (1:2,000 in TBST) next, for 1 h with HRP-conjugated streptavidin 1:200 as we described earlier (Pelletier et al., 1997; Pelletier et al., 2011) Tissue sections were washed in TBST after each incubation and exposed to TBS containing 0.01% H_2_O_2_, 0.05% diaminobenzidine tetrachloride (DAB) (pH 7.7) for 10 min at room temperature (Kabbaj et al., 2001). Controls included the use of the primary antibody alone and, c) the use of the second antibody alone. The sections were mounted in Permount and viewed with a Carl Zeiss Axiophot 2 microscope (Carl Zeiss Canada Ltd., Toronto, ON, Canada) and image capture was carried out with Northern Eclipse software (Empix Imaging Inc. Mississauga, ON, Canada).

#### Data and Statistical Analysis

Analyses were done with Stata software (Stata Corporation, College Station, TX, United States). Data were evaluated with the Student’s t-test or the one-way ANOVA followed by the Tukey honestly significant difference test (THSDT).

## Results

### PCSK9

#### Western Blot Analyses

**FIGURE 1:**
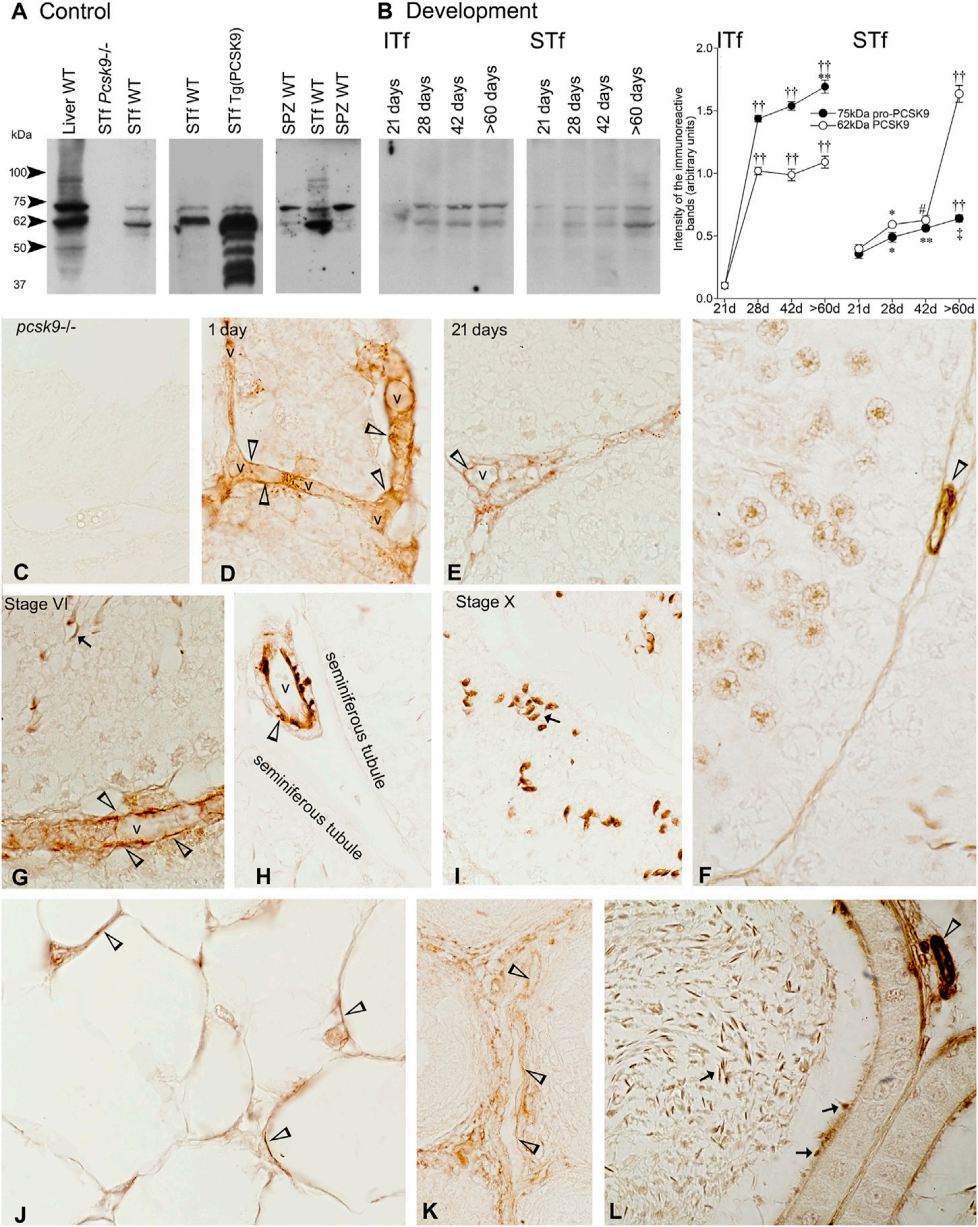
**(A)** PCSK9 protein expression of in mouse testis and epidydimal spermatozoa. *Control*: a 75 kDa pro-PCSK9 and 62 kDa PCSK9 immunoreactive bands accompanied by a 50 kDa band and another around 95 kDa are apparent in the WT mouse liver used as positive control. The *Pcsk9* ^−/−^ mouse seminiferous tubule-enriched fractions (STf) used as negative control bands show no 75 kDa pro-PCSK9 and no 62 kDa PCSK9 bands. The 75 kDa pro-PCSK9 and 62 kDa PCSK9 bands are detected in the seminiferous tubule-enriched fractions (STf) and epidydimal spermatozoa (SPZ); **(B)** Development: In ITf, the increases in pro-PCSK9 and PCSK9 are significant: ^††^ *p* < 0.001 60 days (d) versus (vs.) 21 d and 28 days; 42 d vs. 21 d; 28 d vs. 21 d; **p < 0.005 60 d vs. 28 d; ^††^ *p* < 0.001 60 d vs. 21 d; ^††^ *p* < 0.001 28 d vs. 21 d and ^††^ *p* < 0.001 42 d vs. 21 d. In STf, the increases in pro-PCSK9 and PCSK9 are significant: ^††^ *p* < 0.001 60 days (d) vs. 21 d, ^‡^ *p* < 0.03 60 d vs. 28 d; **p < 0.005 42 d vs. 21 d;*p < 0.05 28 d vs. 21 d; ^††^ *p* < 0.001 60 d vs. 21 d, 28 d and 42 d; ^#^ *p* < 0.02 42 d vs. 21 d and *p < 0.05 28 d vs. 21 d. The values are the mean ± SEM; *n* = 3 per experimental group. **(C–K)** Immunolocalisation of PCSK9: **(C)** *Pcsk9* ^−/−^ mouse testis sections exposed to the PCSK9 antibody showed no reaction product. The stage of the seminiferous epithelium cycle appears at the top of figures. **(D–H)** vessels (v) are PCSK9-positive (open arrowheads). **(G)**, and **(I)** Elongated spermatids (arrows) are labelled; **(J)** Adipocytes in the epididymal fat pad are PCSK-9 positive (open arrowheads). Vessels (v) are labelled in the **(K)** 1-day-old and **(L)** adult mouse epididymis. **(L)** Epididymal spermatozoa are PCSK9-positive (arrows). Original magnification, ×860 **(C, E, G–L)**; X930 **(D,F)**.

The PCSK9 antibody recognized a 75 kDa band corresponding to the uncleaved precursor pro-PCSK9, a 62 kDa band corresponding to the cleaved mature catalytic PCSK9 fragment and a weak 55 kDa immunoreactive band in the wild type (WT) mouse liver used as a positive control (Figure 1A). Neither proPCSK9 nor PCSK9 were detected in the *Pcsk9* ^−/−^ mouse tubule- (Figure 1A) and interstitial tissue-fractions and spermatozoa (not shown). The 75- and 62 kDa PCSK9 were detected in WT mouse seminiferous tubule-fractions where the 62 kDa PCSK9 was the intense band (Figure 1A STf). Unlike tubule-fractions, the 75 kDa PCSK9 band was more intense than 62 kDa band in WT mouse spermatozoa (Figure 1A SPZ). The intensity of the 62 kDa band was increased and the PCSK9-55 kDa form was apparent in the Tg (PCSK9) tubule-fractions (Figure 1A).

### Postnatal Development

#### Interstitial Tissue Fractions

21-day-old mice contained traces of 75 kDa pro-PCSK9 and 62 kDa PCSK9, the levels of both significantly increased in the 28–42- and >60-day-old mice (Figure 1B). The pro-PCSK9 and PCSK9 level profiles were similar though 75 kDa pro-PCSK9 levels were ∼50% higher than 62 kDa PCSK9 levels from 28 to >60 days (Figure 1B).

#### Seminiferous Tubule-Fractions

Pro-PCSK9 and PCSK9 profiles were similar from 21 to 42 days; the level of both significantly increased from 28 days. Thereafter, pro-PCSK9 significantly increased and the PCSK9 levels became threefold higher compared to pro-PCSK9 in >60-day-old-mice (Figure 1B).

### PCSK9 Immunolabeling

The *Pcsk9* ^−/−^ mouse testis exposed to the PCSK9 antibody showed no reaction product (Figure 1C). In WT mice, vessels (Figures 1D–H), the acrosome of the elongated spermatids (Figures 1G,I) and the adipocytes in the epididymal fat pad were PCSK9-positive (Figure 1J). In the epididymis, vessels were labelled in 1- (Figure 1K) and >60-day-old adult mice (Figure 1L). The acrosome of epididymal spermatozoa was labelled (Figure 1L).

### Histological Studies

**FIGURE 2:**
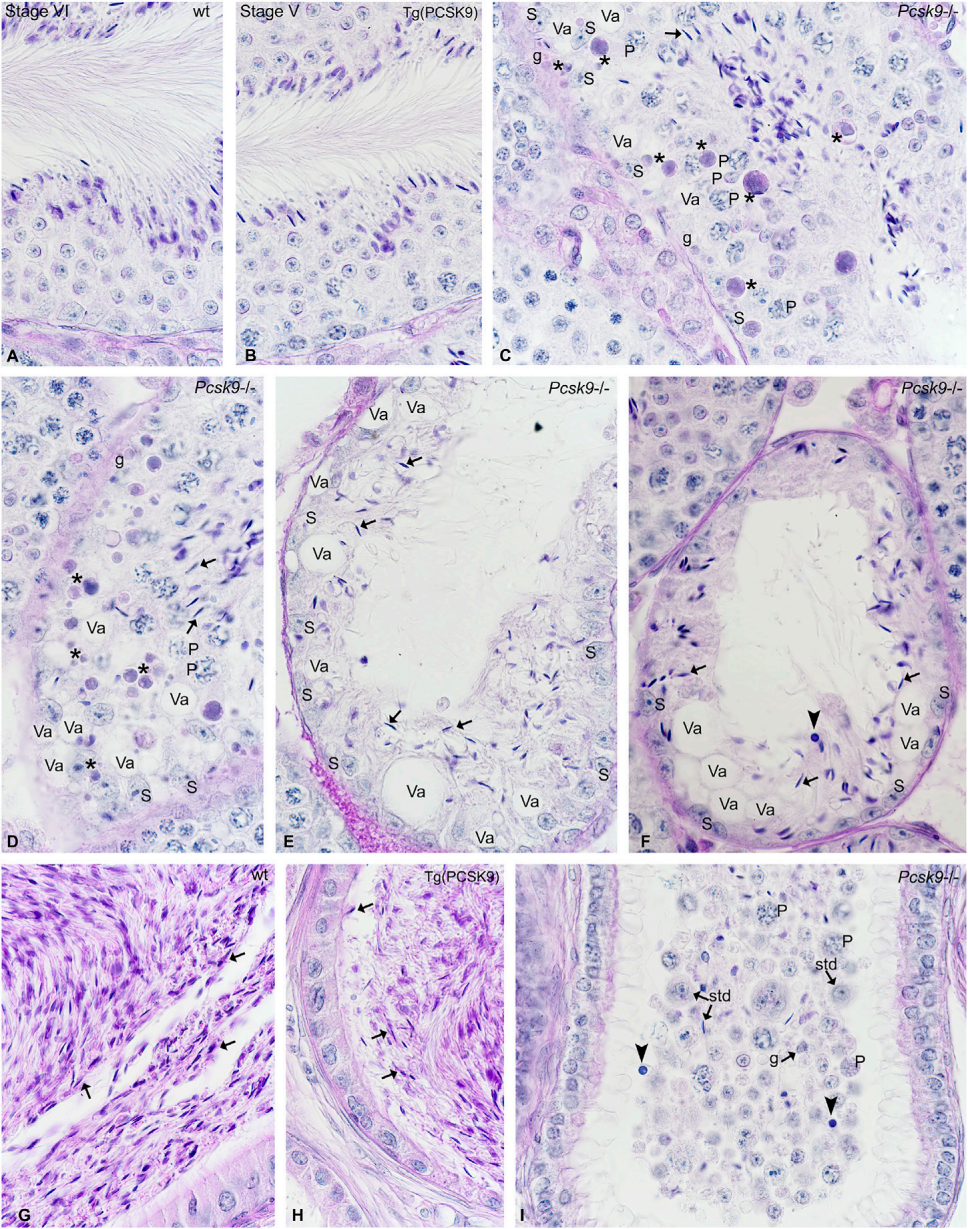
**(A**–**I)** Histological studies in **(A, G)** WT, **(B, H)** Tg (PCSK9) and **(C–F, I)** *Pcsk9* ^−/−^ mouse **(A–F)** testis and **(G–I)** epididymis. WT and Tg (PCSK9) mice show normal spermatogenesis. The stage of the seminiferous epithelium cycle appears at the top of figures. **(C–F)** In *Pcsk9* ^−/−^ mice, the lumen contains cellular debris with elongated spermatids (arrows). Apoptotic figures (asterisks) are plentiful amongst spermatogonia (g) and early spermatocytes. (P: pachytene spermatocytes). Sertoli cells (S) show extensive vacuolisations (Va). The amount of elongated spermatids (arrows), spermatogonia and spermatocytes remaining in seminiferous tubules is reduced. Some tubules contain infiltrated immuno-competent cells (closed arrowhead). **(G–H)** Spermatozoa (arrows) are plentiful in WT and Tg (PCSK9) mouse epididymides. **(I)** The *Pcsk9* ^−/−^ mouse epididymis contain few spermatids (std), pachytene spermatocytes (P) spermatogonia (g) and spermatozoa and immuno-competent cells (closed arrowhead) and debris.

Spermatogenesis was normal in WT (Figure 2A) and Tg (PCSK9) (Figure 2B) mice. A plug of cellular debris occluded the lumen in several tubules in *Pcsk9* ^−/−^ mice (Figure 2C). Sertoli cells showed extensive vacuolisations (Figures 2C–F). Apoptotic figures were plentiful amongst spermatogonia and spermatocytes (Figures 2C,D). The most damaged tubules contained scarce spermatogonia and elongated spermatids with infiltrated immuno-competent cells (Figure 2F). Spermatozoa were plentiful in WT (Figure 2G) and Tg (PCSK9) (Figure 2H) mouse epididymides but few in the *Pcsk9* ^−/−^ mouse head and tail of the epididymis (Figure 2I). A plug of cellular debris including spermatogonia, spermatocytes round and elongated spermatids, and infiltrated leucocytes occupied the lumen in the tubules in the *Pcsk9* ^−/−^ mouse epididymis (Figure 2I).

### Cholesterol

#### Serum

**FIGURE 3:**
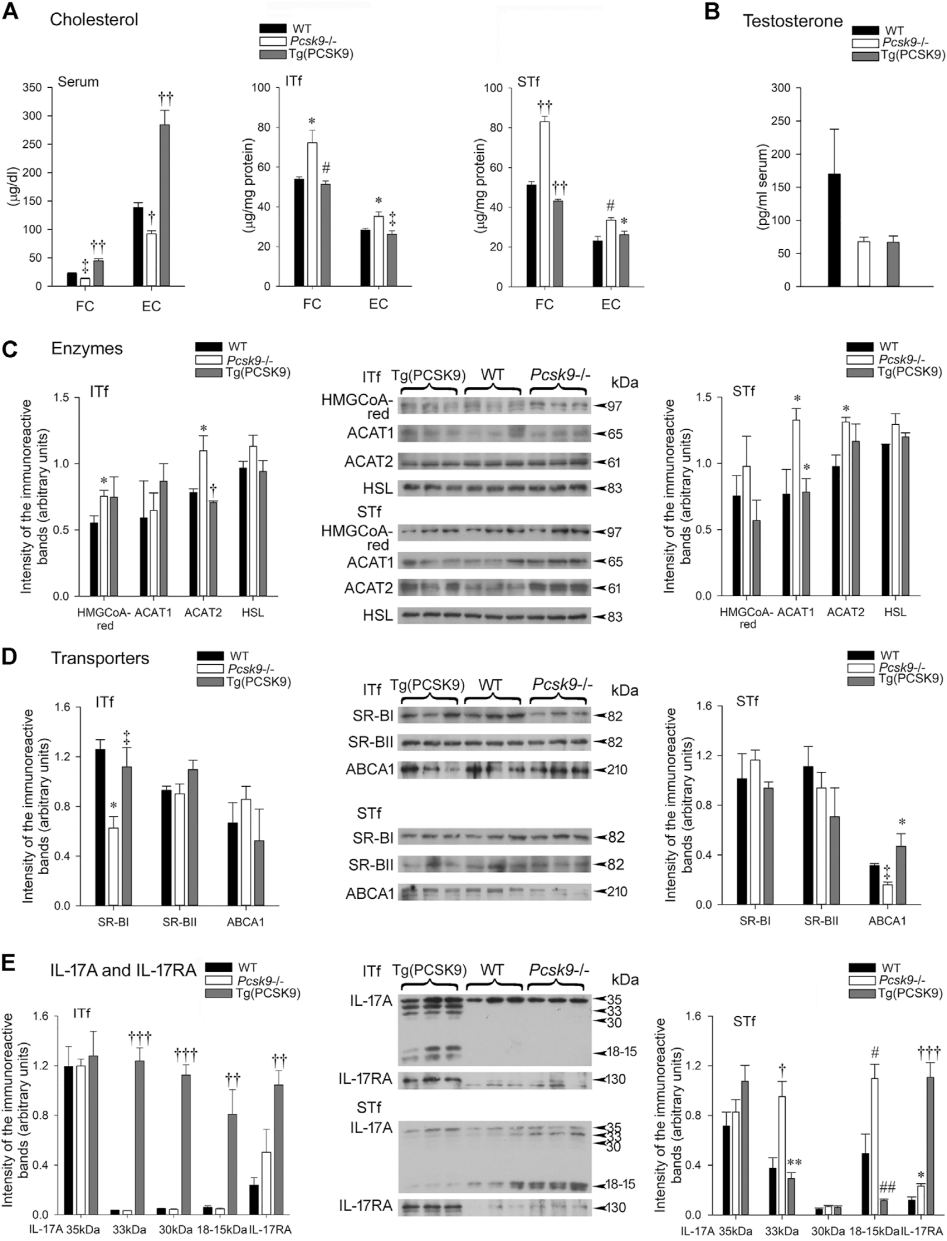
**(A)** Cholesterol **(B)** Serum testosterone **(C)** Cholesterol enzymes **(D)** Cholesterol transporters and **(E)** IL-17A and IL-17RA **(A)** Serum free cholesterol (FC) levels are significantly (^‡^ *p* < 0.03) lowered in *Pcsk9* ^−/−^ compared to the WT counterparts. The increase in Tg (PCSK9) mice is significant (^††^ *p* < 0.001) compared to the WT counterparts and *Pcsk9* ^−/−^ mice. The drop in esterified cholesterol (EC) in *Pcsk9* ^−/−^ is significant (^†^ *p* < 0.01) compared to WT and its increase in Tg (PCSK9) mice is significant (^††^ *p* < 0.001) in comparison to the WT and *Pcsk9* ^−/−^ mice. In ITf, FC increases in *Pcsk9* ^−/−^ (*p < 0.05 vs. WT mice) but recovers in Tg (PCSK9) mice (^#^ *p* < 0.02 vs. *Pcsk9* ^−/−^). EC values significantly increase in *Pcsk9* ^−/−^ mice (*p < 0.05 vs. WT) but decrease in Tg (PCSK9) mice (^‡^ *p* < 0.03 vs. *Pcsk9* ^−/−^). In STf, the increase in FC in *Pcsk9* ^−/−^ mice (^††^ *p* < 0.001 vs. WT mice) and the decrease in Tg (PCSK9) mice are significant (^††^ *p* < 0.001 vs. *Pcsk9* ^−/−^ mice). EC levels significantly augmented in *Pcsk9* ^−/−^ mice (^#^ *p* < 0.02 vs. WT mice) but decreased in Tg (PCSK9) mice (*p < 0.05 vs. *Pcsk9* ^−/−^ mice). **(B)** The differences in testosterone levels are not significant in the WT, *Pcsk9* ^−/−^ and Tg (PCSK9) mice. **(C)** Cholesterol enzymes: In ITf, HMGCoA-red levels increases in *Pcsk9* ^−/−^ (*p < 0.05 vs. WT counterparts). ACAT2 levels increase in *Pcsk9* ^−/−^ (*p < 0.05 vs. WT mice) but drop in Tg (PCSK9) mice (^†^ *p* < 0.01 vs. *Pcsk9* ^−/−^ mice). In STf, ACAT1 levels significantly rise in *Pcsk9* ^−/−^ mice (*p < 0.05 vs. WT mice) but drop in Tg (PCSK9) mice (*p < 0.05 vs. *Pcsk9* ^−/−^ mice). The increase in ACAT2 in *Pcsk9* ^−/−^ mice is significant (*p < 0.05 vs. WT counterparts). **(D)** Cholesterol Transporters: In ITf, SR-BI in *Pcsk9* ^−/−^ mice significantly decreases (*p < 0.05 vs. WT mice) but recovers in Tg (PCSK9) mice (^‡^ *p* < 0.03 vs. *Pcsk9* ^−/−^ mice). In STf, the diminution of ABCA1 in *Pcsk9* ^−/−^ mice is significant (^‡^ *p* < 0.03 vs. WT mice) and its rise in Tg (PCSK9) mice is significant (*p < 0.05 vs. *Pcsk9* ^−/−^ mice). **(E)** IL-17A and IL-17RA: In ITf, the increase in 33- and 30 kDa (^†††^ *p* < 0.0001) and 18–15 kDa IL-17A (^††^ *p* < 0.001) in Tg (PCSK9) mice is significant compared to *Pcsk9* ^−/−^ and WT mice. The rise in IL-17RA Tg (PCSK9) mice is significant (^††^ *p* < 0.001) compared to the WT. In STf, 33- (^†^ *p* < 0.01) and 18–15 kDa IL-17A (^#^ *p* < 0.02) significantly increase in *Pcsk9* ^−/−^ mice compared to the WT counterparts. In Tg (PCSK9) mice, the fall in 33- (**p < 0.005) and 18–15 kDa IL-17A (^##^ *p* < 0.002) is significant compared to *Pcsk9* ^−/−^ mice. The rise in IL-17RA in *Pcsk9* ^−/−^ is significant (*p < 0.05 vs. WT mice). IL-17RA increase in Tg (PCSK9) mice is significant (^†††^ *p* <0.0001 vs. *Pcsk9* ^−/−^ and WT mice). The values are the mean ± SEM; *n* = 3 per experimental group.

Free cholesterol (FC) and esterified cholesterol (EC) levels dropped ∼50% in *Pcsk9* ^−/−^ mice compared to the WT counterparts (Figure 3A). FC and EC levels doubled in Tg (PCSK9) mice compared to the WT mice (Figure 3A) suggesting over expressing the gene had an opposite effect compared to its deletion.

#### Interstitial Tissue and Seminiferous Tubule-Enriched Fractions

Unlike serum cholesterol, FC and EC concentrations significantly escalated in the interstitial tissue- and tubule-fractions (Figure 3A) in *Pcsk9* ^−/−^ compared to WT mice. FC and EC levels were comparable in the Tg (PCSK9) and WT mice (Figure 3A).

### Testosterone

Serum testosterone in *Pcsk9* ^−/−^, Tg (PCSK9) and WT mice showed no significant difference (Figure 3B).

### Enzymes: HMGCoA-Red, ACAT-1,—ACAT-2 and Hormone-Sensitive Lipase

#### Interstitial Tissue-Enriched Fractions

HMGCoA-reductase levels significantly augmented in *Pcsk9* ^*−/−*^ mice not in Tg (PCSK9) mice compared to the WT counterparts (Figure 3C, ITf). ACAT2 protein levels significantly increased in *Pcsk9* ^−/−^ mice compared to WT, whereas Tg (PCSK9) and WT mice had comparable ACAT2 levels (Figure 3C, ITf). In contrast, ACAT1 and HSL levels changed little in *Pcsk9* ^−/−^ and Tg (PCSK9) compared to WT mice (Figure 3C, ITf).

#### Seminiferous Tubule-Enriched Fractions

HMGCoA-reductase and HSL protein contents were not significantly different in Tg (PCSK9), *Pcsk9* ^−/−^ and WT mice (Figure 3C, STf). However, ACAT1 and ACAT2 protein contents significantly increased in *Pcsk9* ^−/−^ mice compared to the WT counterparts (Figure 3C, STf). Tg (PCSK9) and WT mice had comparable ACAT1 and ACAT2 levels (Figure 3C, STf).

### Cholesterol Transporters: SR-BI, SR-BII and ABCA1

#### Interstitial Tissue-Enriched Fractions

SR-BI levels were halved in *Pcsk9* ^−/−^ mice compared to the WT counterparts (Figure 3D, ITf). SR-BI levels were not significantly different in Tg (PCSK9) and WT mice (Figure 3D, ITf). SR-BII and ABCA1 levels in *Pcsk9* ^−/−^ mice were not significantly different in Tg (PCSK9), *Pcsk9* ^−/−^ and WT mice (Figure 3D, ITf).

#### Seminiferous Tubule-Enriched Fractions

SR-BI and SR-BII levels changed little in *Pcsk9* ^−/−^ mice compared to the WT counterparts (Figure 3D, STf). SR-BI and SR-BII levels were not different in Tg (PCSK9) and WT mice (Figure 3D, STf). By contrast, ABCA1 levels dropped 50% in *Pcsk9* ^−/−^ compared to the WT whereas ABCA1 levels were similar in Tg (PCSK9) and WT mice (Figure 3D, STf).

### IL-17A and IL-17RA

#### Interstitial Tissue-Enriched Fractions

Neither IL-17A levels nor IL-17A receptor (RA) levels changed significantly in *Pcsk9* ^−/−^ mice compared to the WT counterparts (Figure 3E, ITf). But in Tg (PCSK9) mice, IL-17RA levels significantly upsurged compared to WT mice despite no changes in IL-17A levels (Figure 3E, ITf).

#### Seminiferous Tubule-Enriched Fractions

Unlike the interstitial tissue-fractions, the 33- and 18–15 IL-17A and IL-17RA levels significantly escalated in *Pcsk9* ^−/−^ compared to the WT mice (Figure 3E, STf). In Tg (PCSK9) mice, 18–15 IL-17A significantly fell whereas IL-17RA levels increased six fold compared to WT mice (Figure 3E, STf).

### LDL-R

#### Western Blot Analyses

**FIGURE 4:**
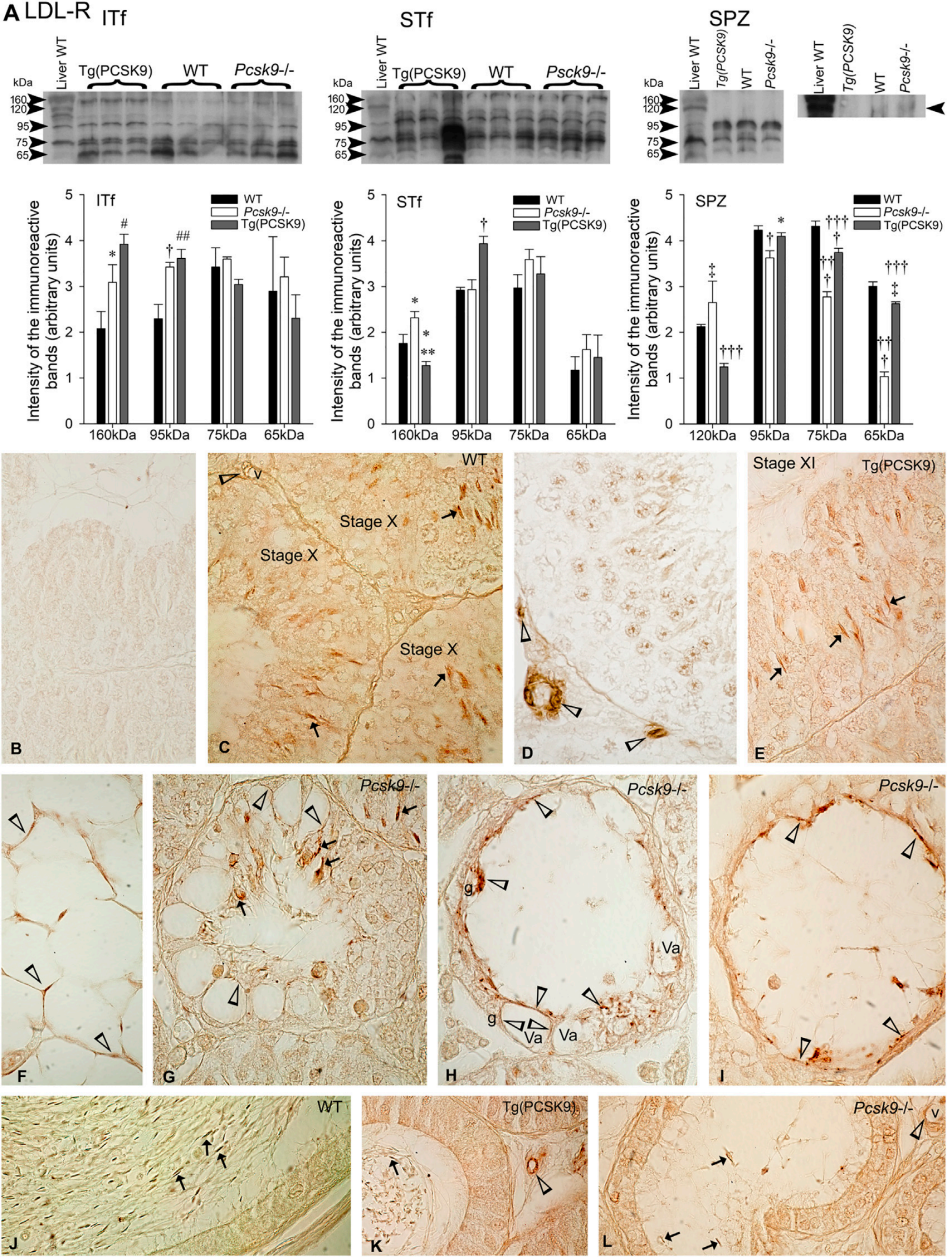
LDL-R **(A)** LDL-R variations in ITf, STf and SPZ in *Pcsk9* ^−/−^, Tg (PCSK9) and WT mice: In ITf, 160- (*p <0.05) and 95 kDa (^†^ *p* < 0.01) LDL-R levels significantly increased in *Pcsk9* ^−/−^ mice compared to WT; in Tg (PCSK9) mice, the increase in 160- (^#^ *p* < 0.02) and 95 kDa (^##^ *p* < 0.002) LDL-R levels is significant compared to WT. In STf, 160 kDa levels significantly increase in *Pcsk9* ^−/−^ mice (∗*p* < 0.05 vs. WT). In Tg (PCSK9) mice, 160 kDa LDL-R significantly drops (**p < 0.005 *vs. Pcsk9* ^−/−^ and *p < 0.05 vs. WT) and the upsurge in 95 kDa levels is significant (^†^ *p* < 0.01) compared to *Pcsk9* ^−/−^ and WT. In SPZ, the increase in 120 kDa levels (^‡^ *p* < 0.03) and the decrease in 95- (^†^ *p* < 0.01), 75- and 65- (^†††^ *p* < 0.0001) are significant compared to WT. In Tg (PCSK9) mice, 120 kDa LDL-R significantly decreases (^†††^ *p* < 0.0001 vs. *Pcsk9* ^−/−^ and WT); the difference in 95 kDa is significant (*p < 0.05 vs. *Pcsk9* ^−/−^); 75 kDa levels significantly differ from *Pcsk9* ^−/−^ (^†††^ *p* < 0.0001) and WT (^†^ *p* < 0.01) and 65 kDa levels differ significantly compared to *Pcsk9* ^−/−^ (^†††^ *p* < 0.0001) and WT (^‡^ *p* < 0.03). The values are the mean ± SEM; *n* = 3 per experimental group. **(B–L)** Immunolocalisation of LDL-R: **(B)** Sections exposed to the second antibody without the first showed no reaction product. The acrosome of elongated spermatids is LDL-R-positive (arrows) in WT **(C)** and **(E)** Tg (PCSK9) mice (arrows). **(C,D)** vessels (v) are LDL-R-positive (open arrowheads). **(F)** Adipocytes in the epididymal pad are labelled (open arrowheads) in WT mice. (**G–I)** Elongated spermatids (arrows) remaining in tubules and vacuoles (open arrowheads) in Sertoli cells are labelled in *Pcsk9* ^−/−^ mice. **(J–L)** Spermatozoa (arrows) and vessels are labelled in the **(J)** WT, **(K)** Tg (PCSK9) and (**L**) *Pcsk9* ^−/−^ mouse epididymides. K and L show LDL-R-positive vessels (v). Original magnification: ×860 **(B–I)**; ×930 **(J,K)**.

The LDL-R antibody detected ∼160-, 95-, 75- and 65 kDa immunoreactive bands in the WT mouse liver used as a positive control (Figure 4A, Liver). The 160- and 95 kDa LDL-R were detected in WT mouse interstitial tissue- and tubule-fractions (Figure 4A, ITf and STf).

#### Interstitial Tissue-Fractions

The 160- and 95 kDa LDL-R levels significantly increased in *Pcsk9* ^−/−^ and Tg (PCSK9) mice compared to WT mice (Figure 4A, ITf).

#### Seminiferous Tubule-Fractions

Only the 160 kDa LDL-R levels significantly increased in *Pcsk9* ^−/−^ mice compared to the WT mice (Figure 4A, STf). Instead in Tg (PCSK9) mice, 160 kDa levels significantly dropped whereas 95 kDa increased compared to the WT mice (Figure 4A, STf).

#### Epididymal Spermatozoa

120 kDa LDL-R levels significantly increased whereas the level of lower molecular weight bands decreased in *Pcsk9* ^−/−^ mice compared to the WT counterparts. In Tg (PCSK9) mice, 120 kDa significantly dropped compared to the WT mice but the levels of the lower molecular weight bands virtually recovered WT values (Figure 4A, SPZ).

### LDL-R Immunolabeling

The WT mice testis sections exposed to the LDL-R antibody without second antibody and/or exposed to the second antibody without the LDL-R antibody showed no reaction product (Figure 4B). The acrosome of elongated spermatids was LDL-R-positive in WT (Figure 4C) and Tg (PCSK9) mice (Figure 4E). In addition, vessels were labelled (Figures 4C,D). LDL-R labelled adipocytes in the epididymal fat pad (Figure 4F). In *Pcsk9* ^−/−^ mice, the acrosome of the scarce elongated spermatids remaining was LDL-R-positive (Figures 4G–I). Sertoli cells exhibited vacuolisations. The vacuoles were lined with LDL-R-positive, the remnants of phagocytosed LDL-R-positive spermatids. Rare spermatogonia remained in tubules (Figures 4G–I). Epididymal spermatozoa and vessels were LDL-R-positive in the WT (Figure 4J), Tg (PCSK9) (Figure 4K) and *Pcsk9* ^−/−^ mice epididymides (Figure 4L).

### Glucose

#### Serum

**FIGURE 5:**
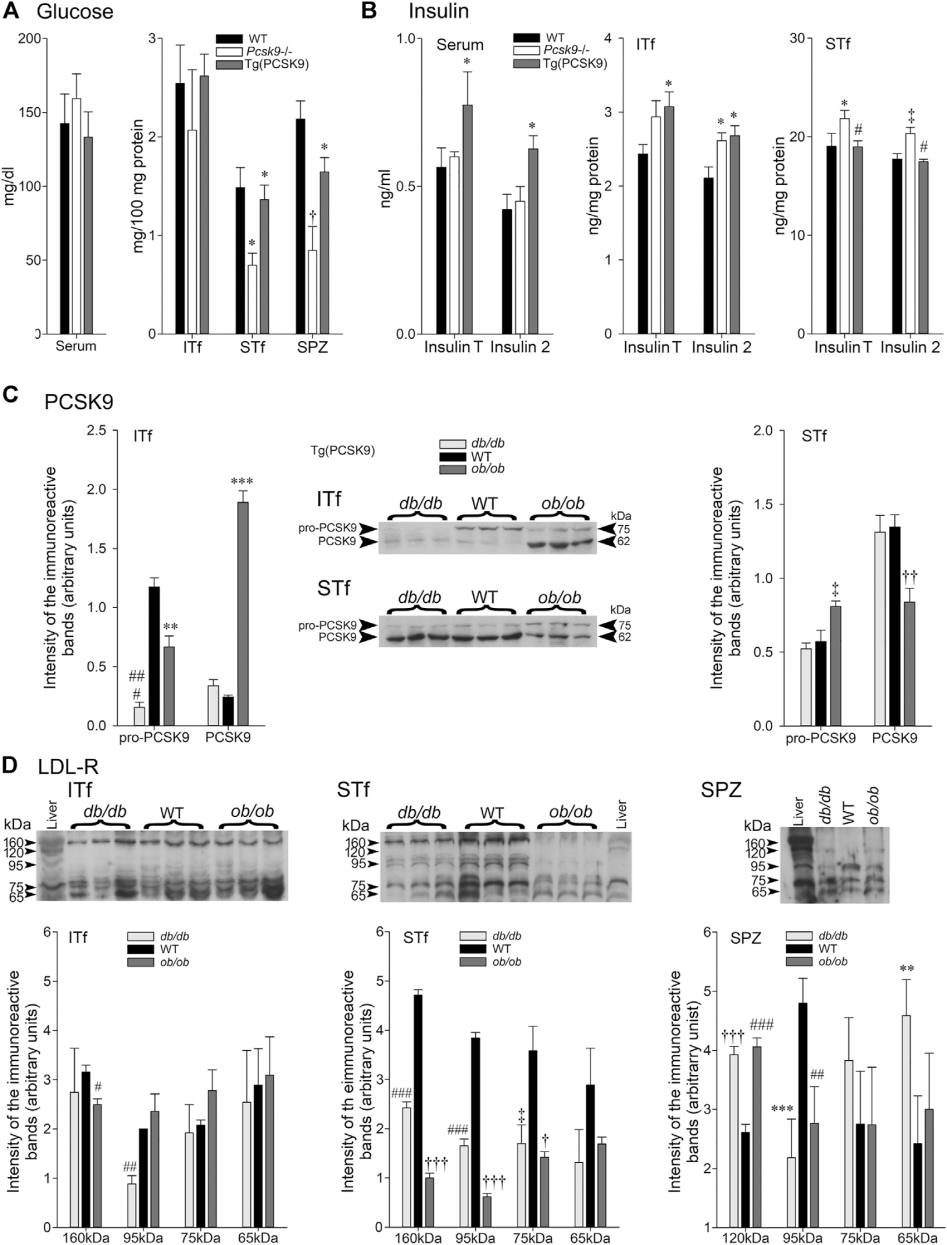
**(A)** Glucose and **(B)** Insulin in *Pcsk9* ^−/−^ and Tg (PCSK9) mice; **(C)** PCSK9 and **(D)** LDL-R in *db/db* and *ob/ob* mice. **(A)** Glucose: *Serum*: glucose concentrations are not significantly different in *Pcsk9* ^−/−^ and Tg (PCSK9) mice compared to WT. In ITf, glucose concentrations are not different in *Pcsk9* ^−/−^, Tg (PCSK9) and WT mice. In STf, glucose levels drop significantly in *Pcsk9* ^−/−^ mice (*p < 0.05 vs. WT) but recover in Tg (PCSK9) mice (*p < 0.05 vs. *Pcsk9* ^−/−^ mice). Glucose concentrations significantly decrease in *Pcsk9* ^−/−^ mouse spermatozoa (^†^ *p* < 0.01 vs. WT mice) but recover in Tg (PCSK9) mice (*p < 0.05 vs. *Pcsk9* ^−/−^). (B) Insulin: Serum insulinT and insulin2 levels mice are significantly higher in Tg (PCSK9) compared to *Pcsk9* ^−/−^ mice and WT (*p < 0.05). ITf: InsulinT significantly increase in Tg (PCSK9) mice (*p < 0.05 vs. WT mice). The upsurge in insulin2 is significant in *Pcsk9* ^−/−^ and Tg (PCSK9) mice (*p < 0.05 vs. WT). STf: insulinT in *Pcsk*9^−/−^ significantly increases (*p < 0.05 vs. WT mice); the WT InsulinT values recover in Tg (PCSK9) mice (^#^ *p* < 0.02). Insulin2 in *Pcsk9* ^−/−^ mice significantly increase (^‡^ *p* < 0.03 vs. WT mice) but recovers WT levels in Tg (PCSK9) mice (^#^ *p* < 0.02 vs. *Pcsk9* ^*−/−*^ mice). The values are the mean ± SEM; *n* = 3 animals per experimental group. **(C)** PCSK9 in *db/db* and *ob/ob* mice. Representative immunoblots with corresponding histograms of pro-PCSK9 and PCSK9 protein levels in ITf and STf. ITf: pro-PCSK9 levels are significantly lowered in *db/db* (^###^ *p* < 0.0002) and *ob/ob* (**p < 0.005) mice compared to WT. PCSK9 levels in *ob/ob* mice significantly increase (***p < 0.0005 vs. WT mice). STf: pro-PCSK9 and PCSK9 significantly increase in *ob/ob* (^‡^ *p* < 0.03 and ^††^ *p* < 0.001, respectively, vs. WT). The values are the mean ± SEM; *n* = 3 animals per experimental group. (D) Representative immunoblots with corresponding histograms of LDL-R protein content in ITf, STf and SPZ in *db/db* and *ob/ob* mice. ITf: The decrease of 160 kDa LDL-R in *db/db* mice is significant (#*p* < 0.0002) compared to WT and the decrease of 95 kDa in *db/db* mice is significant (^##^ *p* < 0 > 002) compared to the WT. STf: 160 kDa LDL-R significantly decreases in *db/db* (^###^ *p* < 0.0002) and *ob/ob* (^†††^ *p* < 0.0001) mice compared to WT. 95 kDa fragment significantly decrease in *db/db* (^###^ *p* < 0.0002) and *ob/ob* (^†††^ *p* < 0.0001) mice compared to WT. 75kDa fragment levels significantly decrease in *db/db* (^‡^ *p* < 0.03) and *ob/ob* (^†^ *p* < 0.01) compared to WT. SPZ: 120 kDa LDL-R levels significantly increase in *db/db* (^†††^ *p* < 0.0001) and *ob/ob* (^###^ *p* < 0.0002) compared to WT. 95 kDa fragment levels significantly drop in *db/db* (***p < 0.0005) and *ob/ob* (^##^ *p* < 0.002) compared to WT. 65 kDa fragment levels are significantly higher in *db/db* (**p <0.005 vs. WT). Values are mean ± SEM; *n* = 3 per group.

Blood glucose concentrations in *Pcsk9* ^−/−^, Tg (PCSK9) and WT counterparts mice did not significantly differ (Figure 5A, Serum).

#### Interstitial Tissue-Fractions

Glucose concentrations were not significantly different in the *Pcsk9* ^−/−^, Tg (PCSK9), and WT counterparts mice (Figure 5A, ITf).

#### Seminiferous Tubule-Enriched Fractions

By contrast, glucose concentrations significantly fell in the *Pcsk9* ^−/−^ mice compared to the WT counterparts (Figure 5A). Tg (PCSK9) and WT mice had similar glucose concentrations (Figure 5A, STf).

#### Epididymal Spermatozoa

Glucose concentrations were significantly lowered in *Pcsk9* ^−/−^ and Tg (PCSK9) compared to the WT mice (Figure 5A, SPZ). Although still lower than WT, glucose levels in Tg (PCSK9) were higher than in *Pcsk9* ^−/−^ (Figure 5A, SPZ).

### Insulin

#### Serum

Total insulin (insulinT) and insulin2 levels were not significantly different in *Pcsk9* ^−/−^ mice and WT counterparts (Figure 5B Serum). Tg (PCSK9) mice had significantly elevated insulinT and insulin2 concentrations compared to the WT mice (Figure 5B, Serum).

#### Interstitial Tissue-Enriched Fractions

InsulinT concentrations showed a tendency to increase though differences were not significant whereas insulin2 significantly augmented in *Pcsk9* ^−/−^ mice in comparison to the WT (Figure 5B, ITf). In Tg (PCSK9) mice, insulinT and insulin2 significantly increased compared to the WT mice (Figure 5B, ITf).

#### Seminiferous Tubule-Enriched Fractions

IinsulinT concentrations were significantly higher in *Pcsk9* ^−/−^ than the WT counterparts (Figure 5B, STf). WT insulinT and insulin2 concentrations were recovered in Tg (PCSK9) mice (Figure 5B, STf).

### PCSK9 in *db/db* and *ob/ob* Mice

#### Interstitial Tissue-Enriched Fractions

The 75 kDa pro-PCSK9 levels dropped six fold whereas the 62 kDa PCSK9 concentration changed little in *db/db* compared to the WT counterparts (Figure 5C, ITf). As well, in *ob/ob* mice, 75 kDa pro-PCSK9 significantly dropped but 62 kDa PCSK9 upsurged eightfold in comparison to the WT (Figure 5C, ITf).

#### Seminiferous Tubule-Enriched Fractions

Neither 75 kDa pro-PCSK9 nor 62 kDa PCSK9 levels in *db/db* mice significantly differ from the WT counterparts (Figure 5C, STf). Conversely, in *ob/ob* mice, pro-PCSK9 levels significantly increased but PCSK9 significantly dropped compared to the WT (Figure 5C, STf).

### LDL-R in *ob/ob* and *db/db* Mice

#### Interstitial Tissue-Enriched Fractions

Only the 95 kDa LDL-R levels significantly dropped in *db/db* mice compared to the WT counterparts. In *ob/ob* mice, 160 kDa LDL-R levels decreased while the levels of the remaining LDL-R bands did not change (Figure 5D, ITf).

#### Seminiferous Tubule-Enriched Fractions

The 160 kDa LDL-R levels and the levels of other breakdown bands significantly decreased in *db/db* and *ob/ob* mice compared to the WT counterparts (Figure 5D, STf).

#### Epididymal Spermatozoa

120- and 65 Da LDL-R levels significantly increased whereas 95 kDa levels significantly fell in *db/db* mice compared to the WT counterparts (Figure 5D). As well, 120 kDa significantly augmented and 95 kDa levels significantly decreased in *ob/ob* mice compared to the WT but the level of the 75- and 65 kDa remained unchanged (Figure 5D, STF).

### Insulin Receptor α-subunit in Pcsk9^−/−^ and Tg (PCSK9) Mice

#### Interstitial Tissue-Enriched Fractions

**FIGURE 6:**
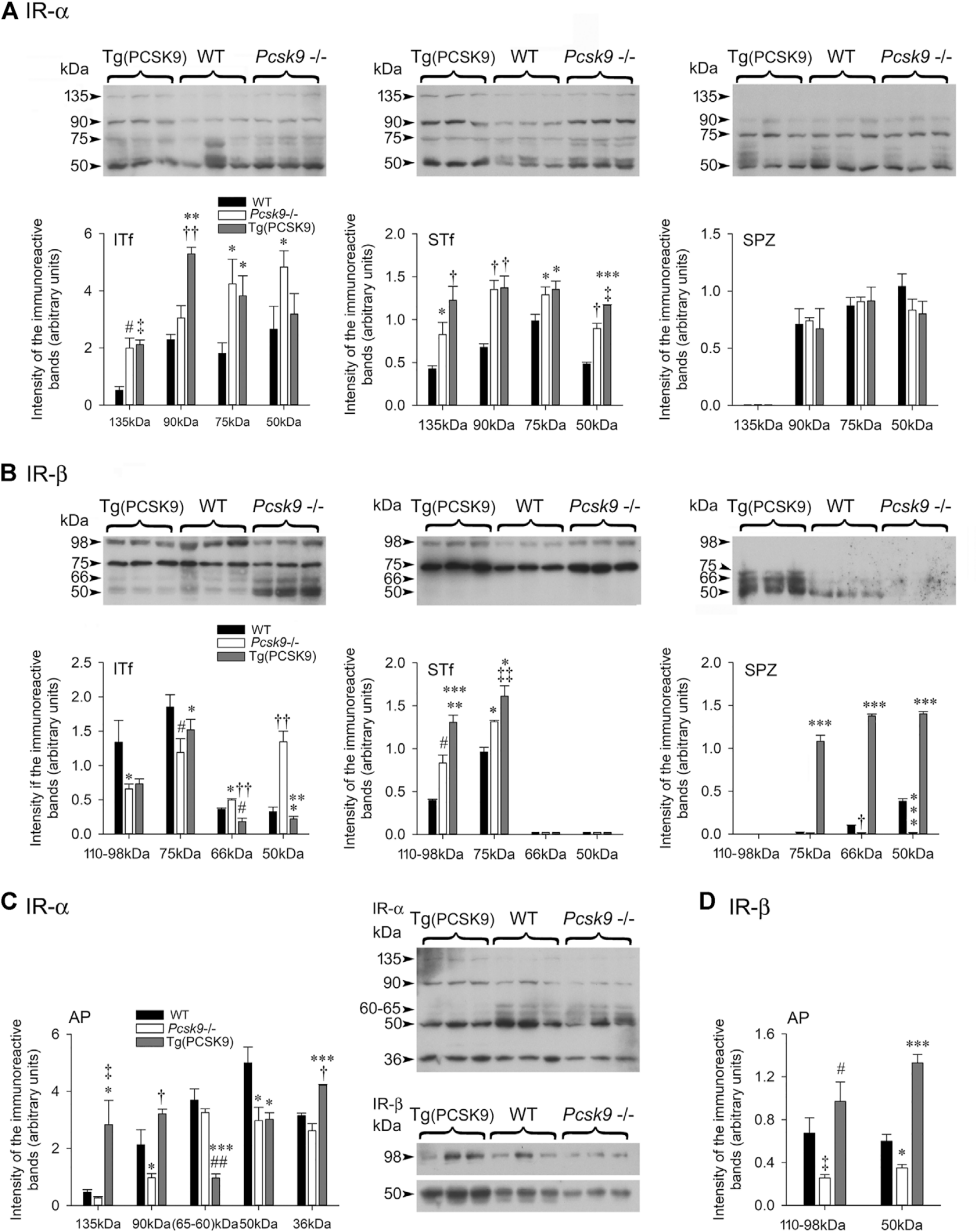
**(A,C)** Insulin receptor (IR)-α and **(B,D)** Insulin receptor (IR)-β subunits in **(A,B)** ITf, STf and SPZ and **(C,D)** anterior pituitary (AP). Representative immunoblots with corresponding histograms of IR subunits α and β protein levels are shown. The values are the mean ± SEM; *n* = 3 per experimental group. **(A)** IR-α subunit: ITf: In *Pcsk9*−/− mice, the increases in 135- (^#^ *p* < 0.02), 75- (*p < 0.05) and 50 kDa (*p < 0.05) IR-α levels are significant compared to WT. In Tg (PCSK9) mice, the 135- (^‡^ *p* < 0.03), 90- (^††^ *p* < 0.001) and 75 kDa (*p < 0.05) IR-α levels are significantly different from WT. In addition, in Tg (PCSK9), 90- (**p < 0.005) and 75 kDa (*p < 0.05) levels are significantly different from *Pcsk9* ^−/−^ mice. STf: the increase in 135- (*p < 0.05), 90- (^†^ *p* < 0.01), 75- (*p < 0.05) and 50 kDa (^†^ *p* < 0.01) IR-α in *Pcsk9* ^−/−^ mice is significant compared to WT. In Tg (PCSK9) mice, the increase in 135- (^†^ *p* < 0.01), 90- (^†^ *p* < 0.01), 75- (*p < 0.05) and 50 kDa (***p < 0.0005) is significant compared to WT mice. 50 kDa fragment levels are also significantly higher in Tg (PCSK9) (^‡^ *p* < 0.03 vs. *Pcsk9* ^−/−^ mice). SPZ: No differences in IR-α levels are observed amongst the experimental groups. **(B)** IR-β subunit: ITf: The differences in 110–98- (*p < 0.05), 75- (^#^ *p* < 0.02), 66- (*p < 0.05) and 50 kDa (^††^ *p* < 0.001) IR-β levels between *Pcsk9* ^−/−^ and WT mice are significant. In Tg (PCSK9) mice, 66- (^#^ *p* < 0.02) is significantly lower compared to WT; 75- (*p < 0.05), and 66- (^††^ *p* < 0.001) and 50 kDa (***p < 0.0005) levels differ significantly from *Pcsk9* ^−/−^ mice. STf: the 110–98- (^#^ *p* < 0.02) and 75 kDa (*p < 0.05) IR-β levels significantly increase in *Pcsk9* ^*−/−*^ mice compared to WT. In Tg (PCSK9) mice, 110–98- (***p < 0.0005) and 75 kDa (^‡‡^ *p* < 0.003) significantly differ from WT and 110–98- (**p < 0.005) and 75 kDa (*p < 0.05) significantly differ from *Pcsk9* ^−/−^ mice. SPZ: In *Pcsk9* ^−/−^, 66 kDa (^†^ *p* < 0.01) and 50 kDa (***p < 0.0005) IR-β levels are significantly lowered compared to WT mice. In Tg (PCSK9) mice, 70-, 66- and 50 kDa (***p < 0.0005) IR-β levels are significantly elevated compared to *Pcsk9* ^−/−^ and WT mice. **(C,D)** Representative immunoblots with corresponding histograms of the IR-α and β subunit protein levels in the anterior pituitary are shown. The values are the mean ± SEM; *n* = 3 per experimental group. IR-α: 90- and 50 kDa IR- α significantly drop in *Pcsk9* ^−/−^ mice (*p < 0.05 vs. WT mice). In Tg (PCSK9) mice, 135- (*p < 0.05), 90- (*p < 0.05), 60–65- (^##^ *p* < 0.002), 50- (*p < 0.05) and 36 kDa (^†^ *p* < 0.01) differ significantly from WT. In addition, 135- (^‡^ *p* < 0.03) 90- (^†^ *p* < 0.01), 60–65- (***p < 0.0005) and 36 kDa (***p < 0.0005) IR-α levels in Tg (PCSK9) mice differ significantly from *Pcsk9* ^−/−^ mice. **(D)** IR-β: 110–98- (^‡^ *p* < 0.03) and 50 kDa (*p < 0.05) IR-β in *Pcsk9* ^−/−^ mice significantly decrease compared to WT. In Tg (PCSK9) mice, 110–98- (^#^ *p* < 0.02) and 50 kDa (***p < 0.0005) IR-β levels significantly increased compared to *Pcsk9* ^−/−^ mice; 50 kDa (***p < 0.0005) IR-β differs significantly from WT.

The antibody detected a ∼135 kDa full length IR-α band and several degradation products of 90-, 75- and 50 kDa (Figure 6A, ITf). The level of 135 kDa and of some breakdown products significantly increased in *Pcsk9* ^−/−^ compared to the WT counterparts (Figure 6A, ITf). In Tg (PCSK9) mice, 135 kDa and most breakdown products significantly augmented compared to the WTs (Figure 6A, ITf).

#### Seminiferous Tubule-Enriched Fractions

The 135 kDa IR-α and most breakdown bands significantly increased in *Pcsk9* ^−/−^ and Tg (PCSK9) mice compared to the WT counterparts (Figure 6A, STf).

#### Epididymal Spermatozoa

The 135 kDa IR-α was not detected in spermatozoa. (Figure 6A, SPZ). The other bands were not significantly different in *Pcsk9* ^−/−^, Tg (PCSK9) and WT.

### Insulin Receptor β-subunit in Pcsk9^−/−^ and Tg (PCSK9) Mice

#### Interstitial Tissue-Enriched Fractions

The antibody detected the 110-98 kDa full length IR-β and 75-, 66- and 50 kDa IR-β degradation products (Figure 6B, ITf). The 110-98 kDa band levels significantly decreased in *Pcsk9* ^−/−^ mice compared to the WT counterparts (Figure 6B, ITf). Full length IR-β levels did not recovered in Tg (PCSK9) mice (Figure 6B, ITf).

#### Seminiferous Tubule-Enriched Fractions

Both, 110–98- and 75 kDa IR-β levels significantly upsurged in *Pcsk9* ^−/−^ and Tg (PCSK9) mice compared to the WT counterparts (Figure 6B, STf).

#### Epididymal Spermatozoa

Mouse epididymal spermatozoa exhibited 50 kDa IR-β and traces of 66- and 75 kDa (Figure 6B, SPZ). The 66- and 50 kDa levels significantly fell in *Pcsk9* ^−/−^ mice compared to the WT counterparts (Figure 6B, SPZ). Conversely, all IR-β band levels significantly upsurged in Tg (PCSK9) mice compared to *Pcsk9* ^*−/−*^ and WT mice (Figure 6B, SPZ).

#### Anterior Pituitary

The 135 kDa full-length IR-α levels were not affected but other low molecular weight band levels significantly decreased in the *Pcsk9* ^−/−^ mice compared to the WT counterparts (Figure 6C, AP). Conversely, in Tg (PCSK9) mice, 135 kDa levels significantly increased in comparison to the WT counterparts (Figure 6C, AP).

The 110–98- and 50 kDa IR-β levels were halved in *Pcsk9* ^*−/−*^ mice compared to the WT counterparts (Figure 6D, AP). By contrast, in Tg (PCSK9) mice, these levels significantly increased compared to *Pcsk9* ^−/−^ and WT mice (Figure 6D, AP).

Table 1 summarizes the changes in cholesterol, enzymes, transporters, IL-17A, IL-17RA, LDL-R, glucose, Insulin Total, Insulin 2, IR-α, IR-β, testosterone in serum, interstitial tissue- and seminiferous tubule-fractions, epididymal spermatozoa and anterior pituitary in *Pcsk9 -/-*, *db/db*, and *ob/ob* mice.

## Discussion

### PCSK9 in Testis and Spermatozoa

Our finding of 75 and 62 kDa PCSK9 in the adult mouse liver agrees with reported similarly sized intracellular PCSK9 bands in the liver (Zaid et al., 2008) and differentiated human adipocytes (Bordicchia et al., 2019). The mature 62 kDa PCSK9 is intra and extracellular whereas 75 kDa pro-PCSK9 which remains in the endoplasmic reticulum, is intracellular (Seidah et al., 2003). 62 kDa PCSK9 regulates the degradation of LDL-R in the extra- and intracellular pathways (Poirier et al., 2009). 75 kDa pro-PCSK9 may also function as a chaperone for LDL-R targeting to degradation prior to its transport to the cell membrane (Schulz and Schlüter, 2017).

**TABLE 1:** An overview of the changes in cholesterol, enzymes, transporters, IL-17A, IL-17RA, LDL-R, glucose, Insulin Total, Insulin 2, IR-α, IR-β, testosterone in serum, interstitial tissue (ITf), seminiferous tubule-fractions (STf), epididymal spermatozoa (SPZ) and anterior pituitary (AP) in *Pcsk9* ^−/−^ mice and in *db/db* and *ob/ob* mice compared to the WT counterparts.

		*Pcsk9* ^*−/−*^				
		Serum	ITf	STf	SPZ	AP
Cholesterol	FC	↓	↑	↑		
	EC	↓	↑	↑		
HMGCoA			↑	=		
ACAT1			=	↑		
ACAT2			↑	↑		
HSL			=	=		
SR-BI			↓	=		
SR-BII			=	=		
ABCA1			=	↓		
IL-17A	35 kDa		=	=		
	18 kDa		=	↑		
IL-17RA			=	↑		
LDL-R	160 kDa		↑	↑		
	120 kDa				↑	
Glucose		=	=	↓	↓	
Insulin Total		=	=	↑		
Insulin2		=	↑	↑		
IR-α	135 kDa		↑	↑		=
	90 kDa		=	↑	=	↓
IR-ß	98 kDa		↓	↑		↓
	75 kDa		↓	↑	=	
	66 kDa		↓	=	↓	
Testosterone		=				

		*db/db*			*ob/ob*		
		ITf	STf	SPZ	ITf	STf	SPZ
pro-PCSK9		↓	=		↓	↑	
PCSK9		=	=		↑	↓	
LDLR	160 kDa	=	↓	↑	↓	↓	↑
	120 kDa

The drop in the pro-PCSK9/PCSK9 ratio in the adult mouse seminiferous tubules suggests enhanced autocatalytic 75 kDa pro-PCSK9 cleavage into 62 kDa PCSK9. The proliferation and differentiation of germ cells are incessant in tubules where the active mature PCSK9 is the predominant form. The autocatalytic cleavage of the inhibitory prodomain of PCSK9 is required for the escape of the proteolytically inactive pro-PCSK9 protein from the endoplasmic reticulum (Seidah et al., 2003; Benjannet et al., 2004) and its passage through the Golgi apparatus (Seidah et al., 2008). These two organelles are extensive in pachytene spermatocytes (Hermo et al., 2010b) and round spermatids (Hermo et al., 2010a). The number of spermatocytes and spermatids augments concurrently with PCSK9 levels between 42 and 60 days in tubules. This could facilitate the autocatalytic cleavage of the inhibitory prodomain of PCSK9 in these germ cells. However, the acrosome of spermatids which differentiate but have ceased to divide was PCSK9-positive in contrast to spermatogonia and spermatocytes that divide but were not labelled. Furthermore, 62 kDa PCSK9 levels increased with the amount of elongated spermatids in tubules suggesting 62 kDa PCSK9 is mainly associated with these cells. The prevalence of 62 kDa PCSK9 in elongated spermatids and the predominance of 75 kDa pro-PCSK9 in spermatozoa entail a change in the activity of the convertase during the transit of gametes in the epididymis. The localization of PCSK9 in the spermatozoon’s acrosome raises the issue of the convertase significance in the sperm-oocyte interaction and fertilisation process. Our immunolocalisation of LDL-R in the acrosome is in line with the report of LDL-R in intracellular membranes associated with enzyme markers of the Golgi apparatus and plasma membranes (Kraemer et al., 1996). The (N- and O-glycosylated) 160 kDa form of LDL-R predominated in tubules whereas non O-glycosylated 120 kDa-LDL-R and fragments prevailed in spermatozoa. The *Pcsk9* ^−/−^ mouse tubules and epididymides showed plentiful apoptotic cells. Spermatogenesis was normal in Tg (PCSK9) mice suggesting that PCSK9 is necessary for germ cell development.

### PCSK9 and Cholesterol Metabolism

Our finding that male *Pcsk9* ^−/−^ mice exhibited lowered serum cholesterol levels confirms an earlier report of hypocholesterolemia in *Pcsk9* deficient mice (Rashid et al., 2005). The cholesterol increase in *Pcsk9* ^−/−^ mouse testis evidences an extra-hepatic effect of PCSK9 on cholesterol homeostasis and uptake in *Pcsk9*−/− mice.

#### Interstitial Tissue

The accumulation of cholesterol concurred with an increase in HMGCoA-red and LDL-R levels and a drop in SR-BI in *Pcsk9* ^−/−^ mice indicating imbalances in cholesterol synthesis and transport. Our immunohistochemical studies localised LDL-R and PCSK9 to the wall of blood vessels. Adding LDL-R molecules at the cell membrane could enhance endocytosis of LDL-cholesterol and increase intracellular cholesterol. Normal cholesterol levels recovered in the interstitium in Tg (PCSK9) though the 160 kDa LDL-R levels remained elevated suggesting that local factors other than PCSK9 are involved in the expression of LDL-R in testicular vessels. Like LDL-R and PCSK9, HSL, ACAT1, and SR-BII also localise to wall of the vessels (Kabbaj et al., 2001; Akpovi et al., 2006, and unpublished results). However, the HSL, ACAT1, and SR-BII protein expression was not affected by the lack of *Pcsk9*.

Our finding that Leydig cells were neither PCSK9- nor LDL-R-immunoreactive agrees with the report of low levels of LDL-R expression in rat Leydig cells (Reaven et al., 2000). Cellular cholesterol is synthesized from acetyl-CoA or up taken from the surroundings. The endogenous cholesterol is used for testosterone synthesis whereas HDL and/or LDL are not the source of cholesterol in Leydig cells (Reaven et al., 2000). In addition, 25-OH-cholesterol contributed by macrophages serves as a substrate for testosterone production in Leydig cells (Nes et al., 2001). The unchanged testosterone levels suggest that the lowering of cholesterol in serum activates several mechanisms for the mobilisation of the cholesterol used in testosterone synthesis in *Pcsk9* ^−/−^ mice. Our finding of increased HMGCoA-red levels in *Pcsk9* ^−/−^ mouse interstitium agrees with the report that endogenous cholesterol is the source of cholesterol for testosterone production (Reaven et al., 2000). The levels of ABCA1, the transporter for the active free cholesterol efflux in Leydig cells, were not affected. Our results indicate a coordination of the synthesis, the uptake and the esterification to regulate cholesterol levels in Leydig cells.

#### Seminiferous Tubules

A part of cholesterol is imported from blood through HDL (Fofana et al., 2000) by multiligand transporters (Akpovi et al., 2006; Pelletier et al., 2009), another part arises from by-products resulting from the phagocytosis of lipid-rich apoptotic germ cells’ membranes remnants and lipid-containing residual bodies (Pelletier and Friend, 1983; Pelletier and Vitale, 1994; Pelletier, 2011). The overload of apoptotic figures in these mice could contribute to the accumulation of cholesterol in tubules in *Pcsk9* ^−/−^ mice. In addition, the higher LDL-R levels in the wall of the vessels could add more cholesterol by its transfer from vessels to cells in tubules. The innumerable vacuoles in Sertoli cells reflect the increased cholesterol levels and lipid droplets dissolved during the dehydration process in alcohols and xylene series.

Sertoli cells and germ cells express SR-BI and SR-BII (Akpovi et al., 2006). The unchanged SR-BI and SR-BII levels suggest that the cholesterol accumulation was not due to passive cholesterol influx in the *Pcsk9* ^−/−^ mouse tubules. Sertoli cells participate in the efflux of cholesterol. Sertoli cells express ABCA1 and *Abca1*-deficient mice exhibit lipid accumulation in Sertoli cells (Selva et al., 2004). The 50% reduction in ABCA-1 in the *Pcsk9* ^−/−^ mouse tubules may contribute to the cholesterol accumulation and suggests that the efflux and reverse cholesterol transport are mediated by ABCA1 in tubules. By contrast, SR-BI is protagonist in the interstitium (Akpovi et al., 2006; Pelletier et al., 2009).

### LDL-R and PCSK9

Our finding of 160 kDa LDL-R and 95-, 75 and 65 kDa proteins in testis agrees with the report of these bands in adipocytes isolated from the rat epidydimal fat pad (Kraemer et al., 1996). A 120 kDa LDL-R immunoreactive band corresponding to the non O-glycosylated form was described in whole testis extracts (Eacker et al., 2008). The smaller sized LDL-R immunoreactive bands are like proteolytic fragments based on the observation that only acetyl-leucine-leucine-methionine (ALLM), an inhibitor of cysteine proteases, prevented the isoproterenol-stimulated proteolysis of LDL-R (Kraemer et al., 1996). The lack of PCSK9 would be expected to lower cholesterol in blood by augmenting the number of LDL-Rs in liver as this was reported in *Pcsk9*-deficient mice (Rashid et al., 2005; Zaid et al., 2008). In agreement with this notion, we found cholesterol concentrations decreased in serum whereas they increased in the *Pcsk9* ^−/−^ mouse testis. The increase in 160- and 95 kDa levels suggests a stimulated LDL-R turnover in the interstitial tissue whereas in tubules, the increased 160 kDa suggests enhanced expression. LDL-R expression is regulated in part by the cholesterol content in testis.

The 120 kDa LDL-R in epididymal spermatozoa represents the immature non O-glycosylated LDL-R. The accumulation of the 120 kDa LDL-R form could reduce LDL uptake in spermatozoa in *Pcsk9* ^−/−^ mice. A 120 kDa membrane-bound C-terminal fragment (CTF) of LDL-R was reported to result from the cleavage of human LDL-R within its extracellular ligand-binding repeats (Banerjee et al., 2019). The 120 kDa CTF LDL-R fragment shows reduced binding capacity and cellular uptake of LDL compared to the full length receptor (Banerjee et al., 2019). The reestablishment of wild type LDL-R values in Tg (PCSK9) mice is evidence that the spermatozoon LDL-R responded to PCSK9 re-expression.

The changes in serum and testicular cholesterol in *Pcsk9* ^−/−^ and *db/db* and *ob/ob* mice are opposite. Serum testosterone and testicular cholesterol levels dropped in *db/db* and *ob/ob* mice (Pelletier et al., 2018). The 75 kDa pro-PCSK9 was less than 62 kDa PCSK9 and total PCSK9 decreased in interstitial tissue in *db/db* mice. The decreased pro-PCSK9/PCSK9 ratio and total PCSK9 may lessen LDL-R degradation though the drop in cholesterol levels does not seem to be due to the PCSK9/LDL-R pathway in the *db/db* mouse interstitium. However in the *ob/ob* mice, the reduction in the pro-PCSK9/PCSK9 ratio was due to a drop in pro-PCSK9 and the eightfold rise in 62 kDa-PCSK9 which led to a reduction in 160 kDa LDL-R. The drop in cholesterol levels may be due to a reduced LDL-R-mediated uptake in *ob/ob* mice.

The *db* mutation lowers total cholesterol in tubules (Pelletier et al., 2018). However, the pro-PCSK9/PCSK9 profile was unaffected though LDL-R decreased in *db/db* mice. By contrast, the *ob/ob* mutation does not change cholesterol content (Pelletier et al., 2018). However, the higher pro-PCSK9/PCSK9 ratio accompanying unchanged total PCSK9 levels suggest a lessened cleavage of the protein and still, LDL-R levels were reduced in *ob/ob* mice. Thus, despite differences in the ratio of FC/EC (Pelletier et al., 2018) and pro-PCSK9/PCSK9 (this paper), LDR-R degradation increased in both mutant mice. The effects of leptin on PCSK9 and lipids in the leptin-deficient *ob/ob* mice plasma are sex-dependent. In serum, leptin suppresses PCSK9 but has no effect on triglycerides or cholesterol in male mice, whereas in female mice, leptin suppresses triglycerides and cholesterol but has no effect on PCSK9 (Levenson et al., 2016).

### PCSK9, Glucose, and Insulin

That the expression of PCSK9 and LDL-R responds to insulin is widely accepted though the conclusions advanced are at variance. Insulin increases LDL-R activity in human hepatoma cells (Wade et al., 1988) whereas it increases the PCSK9 and LDL-R degradation in hepatoma cells and primary hepatocytes (Miao et al., 2015). Insulin increases the degradation of LDL-R in cultured rat adipocytes (Kraemer et al., 1994) whereas it increases LDL-R in fibroblasts (Chait et al., 1979) and mononuclear leukocytes (Krone et al., 1988).

Humans and Guinea pig genomes are said to possess a single copy of insulin-coding gene (Chan et al., 1984). In mouse and rat, the insulin genes are part of a two gene system (Wentworth et al., 1986). In the mouse pancreas, *ins1* and *ins2*, the orthologs to the insulin gene, are transcribed and encode proinsulin peptides (Shiao et al., 2008). Our finding of unchanged glucose and insulinT (total) and insulin2 concentrations in *Pcsk9* ^−/−^ mouse serum agrees with the report that PCSK9 does not alter insulin secretion in mice (Langhi et al., 2009; Peyot et al., 2021).

The mouse interstitial tissue contains both insulinT and insulin2 whereas tubules contain chiefly insulin 2 (Pelletier et al., 2020a). The drop in glucose in presence of elevated insulin2 concentrations evidences a normal response to insulin in tubules in contrast to the interstitial tissue-fractions in which the elevated insulin2 levels were not accompanied by a decrease in glucose in *Pcsk9* ^−/−^ mice. This confirms our earlier report that insulin is glucose-regulated in tubules not in the interstitium (Pelletier et al., 2020b). Moreover, the re-expression of PCSK9 restored glucose and insulin levels in tubules not in the interstitial tissue.

The expression of PCSK9 could be cyclical to respond to the variations in glucose concentrations dictated by the cyclic loss of glucose- and PCSK9-containing spermatids at spermiation in tubules. Serum PCSK9 exhibits a diurnal rhythm synchronous with cholesterol synthesis (Persson et al., 2010). In rodent liver, PCSK9 expression decreases upon fasting and increases after a carbohydrate meal and insulin increases hepatic PCSK9 expression (Costet et al., 2006) whereas glucagon represses PCSK9 (Persson et al., 2009).

The increase in IL-17A and IL-17RA only in tubules suggests that the increase in insulin2 was not due to IL-17A in the interstitium in *Pcsk9* ^−/−^ mice. The serum of diabetic patients and controls show similar IL-17A levels (Roohi et al., 2014). The observation of enhanced glucose tolerance and insulin sensitivity in *Il-17A*-deficient mice lead to the notion that IL-17A contributes to the systemic glucose homeostasis by reducing glucose transporter 4 (GLUT4) (Zúñiga et al., 2010). However, GLUT4 is not present in testis (Kokk et al., 2004).

### Insulin Receptor α and β Subunits

The increase in full length 135 kDa IR-α and IR-α subunit fragments indicates an exacerbated turnover and suggests a gain of active receptor sites for insulin binding in response to the increased levels of the hormone in the *Pcsk9* ^*−/−*^ mouse interstitial tissue and tubules. Conversely, in the anterior pituitary, the decreased IR-α levels could hinder downstream effectors and deregulate the release of hormones. The significance of insulin receptors is evidenced by the report that deleting the encoding gene deregulates the anterior pituitary hormone secretion (Werther et al., 1987).

The drop in the full-length IR-β protein content and the increase in some subunits fragments indicate an increased degradation possibly due to an enhanced internalisation of the receptor in the presence of high insulin2 in the *Pcsk9* ^−/−^ mouse interstitial tissue. By contrast, in tubules, the increase in insulin2 with elevated full-length IR-β and fragment suggests an increased turnover of the receptor. Spermatozoa secrete insulin (Aquila et al., 2005) and express IR-β (Carpino et al., 2010). Gametes responded to PCSK9 since the lack of PCSK9 decreased IR-β levels which recovered in the Tg (PCSK9) mouse spermatozoa.

### PCSK9, IL-17A and Lipid Metabolism

Coordination of the proatherogenic and proinflammatory cytokine IL-17A and IL-17RA protein contents with the synthesis/degradation of lipids differed in the interstitium and tubules in *Pcsk9* ^−/−^ mice. In the interstitial tissue, cholesterol increased concurrently with HMGCoA-red whereas in tubules, the accumulation of cholesterol was concurrent with an increase in IL-17A and IL-17RA levels while the HMGCoA-red protein content remained unchanged. What is known is that changes in ambient cholesterol content activate IL-17A gene transcription (Wang et al., 2015). IL-17A mediates its effect through high cholesterol since lowering cholesterol inhibits cytokine signaling in psoriasis (Varshney et al., 2015). Moreover, PCSK9 helps regulate IL-17A expression since knocking out PCSK9 blocks the increase in IL-17A in *Ldlr*−/− *Apobec1*−/− double knockout mice in serum (Kim et al., 2019). This contrast with the report of no correlation of PCSK9 levels with inflammation markers in human plasma and no response to bacterial endotoxin infusion (Persson et al., 2010). Normal tubule morphology and IL-17A protein contents recovered in Tg (PCSK9) mice suggesting that the deregulation of tubule physiology and high IL-17A were due to the lack of PCSK9 expression.

### PCSK9, IL-17A and Autoimmunity in Testis

The peritubular myoid cells forming the wall of the mouse seminiferous tubules are regulated by mast cell and macrophage products to which they respond by producing factors capable of causing inflammatory changes (Mayerhofer, 2013). The lack of PCSK9 from peritubular capillaries could play a role in the activation of IL-17A-producing cells in testis. The overload of apoptotic germ cells, immune cell infiltrates, sporadic spermatogenic arrest in tubules and depleted spermatozoa accompanying the increase in IL-17A and IL-17RA are pathological features similar to the ones in spontaneous autoimmune orchitis (AIO) reported in humans (Morgan, 1976), dog (Fritz et al., 1976), mink (Tung et al., 1981; Pelletier et al., 2009) and rodents (Furbeth et al., 1989). The abnormal presence of immune cells in tubules and epididymis is evidence that these cells were recruited and suggests the IL-17A activation of macrophages and cytotoxic lymphocytes in *Pcsk9* ^−/−^ mouse testis. The overload of apoptotic germ cells indicates Sertoli cells phagocytic clearance abilities were exceeded. Defects in the regulatory clearance favor the breakdown of self-tolerance during AIO (Pelletier et al., 2009). Self-tolerance, homeostasis, and control of the lymphocyte population are dependent on apoptosis and apoptotic cell uptake in a tissue (Maniati et al., 2008). Increased apoptosis signaling is a response to PCSK9 deficiency. IL-17A induces overexpression of the monocyte chemoattractant protein-1 (MCP-1/CCL2) (Park et al., 2005) in the testicular fluid and cultured testicular macrophages during experimental autoimmune orchitis (EAO) in rats (Guazzone et al., 2003). IL-17A induces early immune responses against infections, participates in autoimmunity and is responsible for destructive inflammatory conditions. IL-17A is produced mainly by T helper cells (Th cells) (Cua and Tato, 2010) and by non-T cells (Rouvier et al., 1993; Kolls and Lindén, 2004). IL17^+^ cells were identified within CD4^+^ and CD8^+^ T cell populations isolated from the rat testis interstitium following EAO (Jacobo et al., 2011). The cholesterol accumulation can create a milieu favoring the switch from non-pathogenic to pathogenic phenotype in IL-17-producing Th17 cells in *Pcsk9* ^−/−^ mouse testis. Th17 cells can induce autoimmunity when activated (Kleinewietfeld and Hafler, 2013). The switch from one phenotype to the other is determined by the lipid metabolism (Wang et al., 2015) and cytokines (Bettelli et al., 2006). Both were altered in *Pcsk9* ^−/−^ mouse testis.

## Conclusion

The lack of PCSK9 expression produced opposite effects in the serum and testis. Deleting *pcsk9* augmented cholesterol and insulin2 concentrations in testis but decreased glucose in tubules and spermatozoa. Furthermore, LDL-R augmented in testis and spermatozoa. The accumulation of cholesterol was concomitant with an overload of apoptotic cells, defects in the Sertoli cell’s regulatory clearance indicating the breakdown of self-tolerance, infiltration of immune cells which have the capacity to activate IL-17A-producing cells and increased IL-17A and IL-17RA protein contents associated with autoimmunity. The action of IL-17A requires high cholesterol content. The accumulation of cholesterol resulting from the PCSK9 deficiency creates a milieu favorable to a change in the phenotype of the IL-17-producing Th17 cells into pathogenic cells, the development of inflammatory conditions and self-tolerance breakdown in testis. The recovery of spermatogenesis and normal testicular histology in Tg (PCSK9) mice indicates the lack of PCSK9 influences testicular function and could reduce fertility in mice. IL-17A and insulin are implicated in the deregulation of spermatogenesis and capacity of Sertoli cell to recycle apoptotic cells. Our results show that lipid and glucose metabolism with immunotolerance contribute to the normal physiology of the testis.

## Data Availability

The original contributions presented in the study are included in the article/supplementary material, further inquiries can be directed to the corresponding author.
